# Identification of loci associated with water use efficiency and symbiotic nitrogen fixation in soybean

**DOI:** 10.3389/fpls.2023.1271849

**Published:** 2023-11-16

**Authors:** Muhammad Arifuzzaman, Sujan Mamidi, Alvaro Sanz-Saez, Hossein Zakeri, Andrew Scaboo, Felix B. Fritschi

**Affiliations:** ^1^ Division of Plant Science and Technology, University of Missouri, Columbia, MO, United States; ^2^ HudsonAlpha Institute for Biotechnology, Huntsville, AL, United States; ^3^ Department of Crop, Soil and Environmental Sciences, Auburn University, Auburn, AL, United States; ^4^ College of Agriculture, California State University-Chico, Chico, CA, United States

**Keywords:** carbon isotope ratio, nitrogen isotope ratio, genome-wide association mapping, whole genome resequencing, water use efficiency

## Abstract

Soybean (*Glycine max*) production is greatly affected by persistent and/or intermittent droughts in rainfed soybean-growing regions worldwide. Symbiotic N_2_ fixation (SNF) in soybean can also be significantly hampered even under moderate drought stress. The objective of this study was to identify genomic regions associated with shoot carbon isotope ratio (δ^13^C) as a surrogate measure for water use efficiency (WUE), nitrogen isotope ratio (δ^15^N) to assess relative SNF, N concentration ([N]), and carbon/nitrogen ratio (C/N). Genome-wide association mapping was performed with 105 genotypes and approximately 4 million single-nucleotide polymorphism markers derived from whole-genome resequencing information. A total of 11, 21, 22, and 22 genomic loci associated with δ^13^C, δ^15^N, [N], and C/N, respectively, were identified in two environments. Nine of these 76 loci were stable across environments, as they were detected in both environments. In addition to the 62 novel loci identified, 14 loci aligned with previously reported quantitative trait loci for different C and N traits related to drought, WUE, and N_2_ fixation in soybean. A total of 58 *Glyma* gene models encoding for different genes related to the four traits were identified in the vicinity of the genomic loci.

## 1 Introduction

Drought limits soybean [*Glycine max* (L.) Merr.] productivity by affecting diverse processes during reproductive (Sinha et al., 2021) and vegetative growth, including photosynthesis and biological nitrogen fixation (Kunert et al., 2016; Martynenko et al., 2016; Zhu et al., 2016; Dubey et al., 2019). Limitations in water availability to sustain crop growth and grain yield are expected to increase further in many regions of the world (Luo et al., 2019), amplifying the urgency to develop varieties with superior performance under water deficit stress conditions. However, given low heritability and complex scenarios such as timing, duration, and intensity of water deficit stress imposition, selecting for increased yield or maintenance of yield under drought stress is challenging for breeding programs (Bhat et al., 2016). Exploration of the genetics underpinning physiological traits that influence and/or are responsive to water deficit stress can provide insights that can be leveraged to develop more drought-tolerant cultivars (Sallam et al., 2019; Xiong et al., 2021).

Phenotyping of physiological traits in large populations grown under field conditions can be difficult, but, tissue C and N stable isotope analysis are suitable for such scenarios and can be used to assess water use efficiency (WUE) and symbiotic N_2_ fixation (SNF), two traits which can be selected to enhance crop drought tolerance (Condon et al., 2002; Rebetzke et al., 2002; Richards et al., 2002). Vadez and Ratnakumar (2016) recently demonstrated that peanut (*Arachis hypogea*) cultivars with high WUE, measured using mini-lysimeters, produced higher yields than cultivars with low WUE. However, for most large-scale breeding programs, effective screening for WUE using lysimeters or other time-consuming tools is not feasible (Hu and Xiong, 2014). Isotopic carbon composition, expressed either as carbon isotope discrimination (CID) or carbon isotope ratio (δ^13^C), is physiologically linked to leaf and whole plant WUE (Farquhar et al., 1989; Ehleringer et al., 1991; Easlon et al., 2014). Isotopic carbon composition is a useful surrogate measure of WUE in several crop species, namely, wheat (*Triticum aestivum*) (Condon et al., 2002; Rebetzke et al., 2002), barley (*Hordeum vulgare*) (Çağirgan et al., 2005), peanut (Hubick et al., 1986), cowpea (*Vigna unguiculata*) (Hall et al., 1990), common bean (*Phaseolus vulgare*) (Sanz‐Saez et al., 2019), and soybean (White et al., 1996), and has been used in breeding programs to develop more drought tolerant wheat in Australia (Condon et al., 2002; Rebetzke et al., 2002). Previous studies with soybean successfully identified numerous loci associated with δ^13^C based on diversity panels (Dhanapal et al., 2015a; Kaler et al., 2017; Steketee et al., 2019) and biparental mapping populations (Specht et al., 2001; Bazzer et al., 2020a; Bazzer et al., 2020b) using 35,234 or fewer single-nucleotide polymorphism (SNP) markers.

Symbiotic N fixation provides an important advantage for soybean production compared to other grain crops, as it eliminates the need for N fertilization in most production environments under standard management practices. Up to 94% of the total N requirement by the plant can be acquired through SNF in N-deficient soil (Harper, 1987). However, despite the significant capacity for SNF, N availability can limit soybean yields under a range of environmental conditions, including drought (King and Purcell, 2001; Purcell et al., 2004; King and Purcell, 2006; King et al., 2014; Purcell, 2015), high soil temperature (Lindemann and Ham, 1979; Montañez et al., 1995), flooding stress (Pasley et al., 2020), and soil salinity (Elsheikh and Wood, 1995). SNF is highly sensitive to drought stress and this sensitivity can result in significant yield reductions under water-limiting conditions (Durand et al., 1987; Sinclair et al., 1987; Sall and Sinclair, 1991; Purcell et al., 1997). Sinclair et al. (2010) modeled the impacts of changes in selected morpho-physiological traits on soybean yields across much of the U.S. soybean production region and found that reducing SNF sensitivity to drought would result in the greatest yield benefit among the examined traits. These and other studies highlight the need and potential for improvements in SNF to enhance soybean productivity.

Genotypic variation in SNF under normal and water-limited conditions has been well documented, revealing opportunities to improve soybean by selecting genotypes with enhanced SNF and, in particular, genotypes capable of sustained SNF under water-limited conditions (King et al., 2014). This is exemplified by the success of Chen et al. (2007), who developed soybean varieties with enhanced drought tolerance by breeding with genotypes that maintain high SNF under water-limited conditions. To better understand the genetics and facilitate breeding for elevated and sustained SNF, several research groups have conducted studies to identify quantitative trait loci (QTLs) for SNF and/or SNF-associated traits in numerous legumes (e.g., Heilig et al., 2017; Kamfwa et al., 2019;, Bourion et al., 2010; Ohlson et al., 2018). In soybean, studies have explored QTL for several nodulation traits (Santos et al., 2013; Hwang et al., 2014; Yang et al., 2019; Zhu et al., 2019; Ni et al., 2022), ureide accumulation (Hwang et al., 2013; Ray et al., 2015a), N isotope ratio (δ^15^N), (Steketee et al., 2019; Bazzer et al., 2020c), and percent N derived from the atmosphere (Ndfa) (Dhanapal et al., 2015b; Liyanage et al., 2023).

δ^15^N is a measure of the relative abundance of ^14^N and ^15^N isotopes in plant tissue relative to their relationship in air and is expressed in ‰. Because soils have a higher ^15^N abundance than air, SNF enriches ^14^N relative to ^15^N in tissues of N-fixing plants, which consequently exhibit a lower δ^15^N compared to plants that rely on soil mineral N uptake for their N supply (Shearer and Kohl, 1986). Therefore, δ^15^N can be used directly to compare the relative SNF of different genotypes (Steketee et al., 2019; Bazzer et al., 2020c) and to calculate the percent Ndfa when a genotype that depends only on soil mineral N as a N source is used as a reference (Kohl and Shearer, 1981). An advantage of the N stable isotope technique is that it can be used to assess large populations grown under field conditions and provides an integrated signal of relative SNF over the course of plant growth.

It is well established that leaf N content is closely related to net photosynthesis, N uptake is closely related to soybean yield, and N availability can limit soybean yields in a broad range of environments (Sinclair and Horie, 1989; Rotundo et al., 2014; Cafaro La Menza et al., 2017; Cafaro La Menza et al., 2020). Pazdernik et al. (1997) and Rotundo et al. (2014) showed that yields of elite soybean cultivars were more closely related with total N uptake (determined based on shoot analysis) than with total shoot biomass. As one of the factors in determining total N uptake, shoot tissue [N] concentration is, thus, of great relevance. Previously, King and Purcell (2006) showed that genotypes with inherently lower shoot [N] were able to maintain SNF compared to genotypes with inherently higher shoot [N]. Of course, N assimilation is intimately intertwined with C assimilation, and CN-dynamics are closely regulated by complex mechanisms (Djekoun and Planchon, 1991). In soybean, these complex interactions also extend to SNF, which, for example, is more sensitive to drought than net photosynthesis (Djekoun and Planchon, 1991), and partial recovery from prolonged drought is slower for SNF than for photosynthesis (Djekoun and Planchon, 1991). Thus, the characterization of genotypic variation and mapping of shoot [N] and shoot carbon nitrogen ratio (C/N) ratio in conjunction with SNF and WUE can provide valuable information for soybean germplasm improvement. Indeed, in addition to studies mentioned above that have identified loci for δ^13^C and δ^15^N or Ndfa, previous research has also identified markers for soybean shoot [N] and/or C/N ratio (Hwang et al., 2013; Dhanapal et al., 2015b; Steketee et al., 2019).

δ^13^C (WUE), δ^15^N (SNF), [N], and C/N ratio are quantitative in nature and controlled by many genes with minor effects (Adiredjo et al., 2014; Dhanapal et al., 2015a; Dhanapal et al., 2015b; Kaler et al., 2017). Molecular markers and QTL associated with these traits can facilitate marker-assisted selection and genomic prediction and accelerate the breeding process. Previous soybean genome-wide association (GWA) studies identified genomic regions and markers associated with δ^13^C, δ^15^N, [N], and C/N ratio based on a limited number of molecular markers [12,347 SNP by Dhanapal et al. (2015a); 31,145 SNP by Dhanapal et al. (2015b); 31,260 SNP by (Kaler et al. (2017); and 35,262 SNP by (Steketee et al. (2019)] derived from the SoySNP50K iSelect Beadchip (Song et al., 2013). In the current study, approximately 4.1 million SNP markers derived from whole-genome resequencing of 105 genotypes were used to conduct GWA mapping for δ^13^C, δ^15^N, [N], and C/N ratio. Whole-genome resequencing allows the detection of a large number of molecular markers, such as SNPs and Insertion-Deletions (Indels) in crop species (Xu and Bai, 2015; Goodwin et al., 2016; Torkamaneh et al., 2018), and high genome-wide marker density increases the precision of marker-trait associations (Huang et al., 2009; Xu et al., 2013; Xu and Bai, 2015). The main objective of this study was to utilize the high-resolution marker density derived from whole-genome resequencing to identify molecular markers and genomic regions associated with δ^13^C, δ^15^N, [N] and C/N traits in a diversity panel consisting of 105 soybean genotypes and to explore these regions for candidate genes that may control these traits.

## 2 Materials and methods

### 2.1 Plant material and field experiments

Genotypes included in this study were selected because of available whole-genome resequencing data and the desired focus on maturity group (MG) II and III germplasm. The 105 diverse genotypes (**Supplementary Table S1**) were composed of 41 in MG II, 60 in MG III, and four in MG IV. Seeds, originally obtained from the United States Department of Agriculture (USDA) Soybean Germplasm Collection, were increased in 2013. Experiments were conducted at the Bradford Research Center near Columbia, Missouri, on a Mexico silt loam (fine, smectitic, mesic Vertic Epiaqualfs) soil. Seeds were sown on 6 May 2014 and on 5 May 2016 at a density of 34.4 seeds m^−2^ in 3-m long single-row plots that were spaced 0.76-m apart. The experiment established in 2015 had to be terminated due to inadequate emergence caused by adverse environmental conditions. Entries were arranged in a randomized complete block design with three replications. Due to the long history of soybean production at that location, inoculation with *Bradyrhizobium diazoefficiens* was omitted. Weeds were controlled with pre- (Dual II Magnum; S-metolachlor at 1.68 kg ha^−1^) and post-emergence (Basagran, Sodium salt of bentazon at 0.45 kg ha^−1^ ai., and Fusilade DX, Fluazifop-P-butyl at 0.425 kg ha^−1^ ai.) herbicide applications, which were complemented with manual weeding. Experiments were conducted under rainfed conditions without supplemental irrigation. Rainfall and temperature data were recorded by a weather station located within 500 m of the field sites and downloaded from the Missouri Historical Agricultural Weather Database (http://agebb.missouri.edu/weather/history/).

### 2.2 Phenotyping for stable isotope and C and N concentrations

Shoot biomass of five representative plants from each plot was harvested at the beginning of bloom to full bloom [R1 to R2; (Fehr et al., 1971)] stages in both years. Due to poor stand densities of 10 entries, only samples from 95 genotypes were collected in 2016. Samples were dried in a forced-air drier at 60°C until constant weight. Dry samples were ground (Thomas Model 4 R Mill, Thomas Scientific, NJ, USA) to pass a 2-mm screen, subsampled and processed with a cyclone mill (UDY Corporation, CO, USA) to pass a 1-mm screen, and in a third step, ball milled in 15-ml tubes with a 9.52-mm stainless steel ball (440C Stainless Steel Ball, Abbott Ball Company, Inc., CT, USA) in a Geno/Grinder (SPEX CeertiPrep, Inc., NJ, USA). Three milligram of the ball-milled sample from each plot was loaded into tin capsules (Costech Analytical Technologies Inc., CA, USA) and sent to the University of California Stable Isotope Facility in Davis, CA (https://stableisotopefacility.ucdavis.edu/carbon-and-nitrogen-solids) for analysis with an isotope ratio mass spectrometer (IsoPrime, Elementar France) coupled to an elemental analyzer (EA3000, EuroVector). The δ^13^C and δ^15^N of each sample were expressed relative to the international standards V-PDB (Vienna Pee Dee Belemnite) and air, respectively, according to the following equations (O’Leary, 1981; Mariotti, 1983; Sharp, 2017):

Carbon isotope ratio:


(i) $$\delta^{13}\text{C }(‰)\;=\;1000\;\left(R_{\text{x}}–R_{\text{VPDB}}\right)/R_{\text{VPDB}}$$

where *R* is the ratio of the heavy to the light isotope (^13^CO_2_/^12^CO_2_) and *R*_x_ and *R*_VPDB_ are the isotope ratios of the sample and the standard, respectively.

Nitrogen isotope ratio:


(ii) $$\delta^{15}\text{N }(‰)\;=\;1000\;\left(R_{\text{x}}–R_{\text{airN}2}\right)/R_{\text{airN}2}$$

where, *R* is the ratio of the heavy to the light isotope (^15^N/^14^N) and *R*_x_ and *R*_airN2_ are the isotope ratios of the sample and the standard, respectively.

Carbon and N concentrations were determined as part of the stable isotope analysis with the elemental analyzer (EA3000, EuroVector). The C and N concentrations of each sample were expressed as mg C or N g^−1^ dry weight. Sample C to N ratios (C/N) was calculated by dividing the C concentration by the N concentration.

### 2.3 Statistical analysis of phenotypic data

The two years, 2014 and 2016, were treated as two different environments. Analysis of variance (ANOVA) was performed in SAS 9.4 (SAS Institute Inc., Cary, NC, USA) for each year and across the two years using the PROC MIXED procedure. In individual-environment ANOVA, genotype was treated as a fixed effect, and replications within an environment were treated as random (Bondari, 2003). In across-environment ANOVA, all factors were treated as fixed effects, except replications within environments that were considered random effects (Bondari, 2003). The Pearson correlation coefficient between the traits within and across the environments was calculated in SAS 9.4 using PROC CORR. Entry-mean basis broad sense heritabilities were calculated as *H* ^2^ = σ^2^_g_/[σ^2^_g_ + σ^2^_ge_/k + σ^2^_e_/(rk)], where σ^2^_g_ is the genotypic variance, σ^2^_ge_ is the genotype by environment interaction variance, k is the number of environments, r is the number of replications (Holland et al., 2003). The variance components were estimated using the PROC VARCOMP procedure in SAS 9.4 with the restricted maximum likelihood estimation method following Holland et al. (2003).

The best linear unbiased predictions (BLUP) were calculated for each trait for each year using PROC MIXED functions in SAS 9.4 (SAS Institute Inc., USA), where all factors were treated as random factors (Robinson, 1991; Piepho et al., 2008). Calculated BLUP values were used for GWA mapping.

### 2.4 Genotypic data, SNP identification, and filtering

Whole-genome resequencing (15X and 40X coverage depending on genotype) was conducted by others, and the information was deposited in Soykb [Liu et al. (2016b); http://soykb.org/NGS_Resequence/NGS_index.php]. SNPs were identified following the PGen workflow described by (Liu et al., 2016b). A total of 11,972,496 raw SNP markers were obtained for the 105 genotypes. Monomorphic markers and markers with 50% missing data were filtered out, and missing data for the remaining markers were imputed using BEAGLE 5.1 with default parameters (Browning et al., 2018). Finally, markers with less than 5% minor allele frequency were filtered out, resulting in 4,108,002 SNP that were used for GWAM.

### 2.5 Genome-wide association analysis

GWA mapping was performed by implementing the Fixed and Random Model Circulating Probability Unification (FarmCPU) model in R (Liu et al., 2016a). FarmCPU performs multi-locus mixed model in two parts, the fixed effect model and random effect model, and uses them iteratively until there is no change between them in terms of identified markers. Principle component analysis was conducted using PLINK 1.9 (Purcell et al., 2007) with a subset of 50,000 LD pruned markers. PCs that cumulatively explain 25% of the total variation of the population were added as covariates in the FarmCPU model to control for the population structure.

As the Bonferroni *p*-value threshold to declare a marker significantly can be too stringent and exclude some true associations resulting in false negatives (Moghaddam et al., 2016; Arifuzzaman et al., 2019; Kaler et al., 2020), the *p*-value thresholds determined by using “FarmCPU *p-*value threshold” function with 100 permutations were used. This function breaks the relationships of the phenotypes with the genotypes through permutation and suggests a suitable *p*-value threshold for the respective trait (Liu et al., 2016a). As the phenotypic distribution of each trait differs, the FarmCPU *p*-value threshold function generates different *p*-value thresholds for different traits. In addition to all markers meeting the stringent cutoff *p*-value thresholds determined by the FarmCPU threshold function, results were also inspected for markers that passed a -log (*p*-value) threshold of 3.5 for each trait. Markers identified based on this threshold were considered significant either if they were detected in both years or detected in one of the two years and were within 1.5 Mb upstream or downstream of a locus for the respective trait identified in the previous studies (Dhanapal et al., 2015a; Dhanapal et al., 2015b; Kaler et al., 2017; Steketee et al., 2019; Bazzer et al., 2020a; Bazzer et al., 2020b; Bazzer et al., 2020c).

Stepwise regression procedure was implemented in R statistical software (function “*step*”) to identify the minimal number of markers independently associated with a particular trait in each year (Meyer et al., 2013; Gurung et al., 2014; Mamidi et al., 2014; Delaneau et al., 2017). The stepwise regression procedure accounts for QTL × QTL interactions and retains only the major and independent QTL (Delaneau et al., 2017). Significant markers with stringent cutoffs and those identified in both years with a cutoff of -log (*p*) = 3.5 were included in the stepwise procedure. In this procedure, both the marker and the model needed to be significant at *P* < 0.05 for the stepwise inclusion of a marker. Genome-wide LD blocks were estimated using the “BigLD” function in the gpart R package (Kim et al., 2019) with an LD cutoff of 0.7. If multiple significant markers were found within the same LD block, they were tagged as one locus. Otherwise, single markers represent the anchoring markers of the respective LD region.

### 2.6 Candidate genes

All gene models within the LD block of each significant marker were extracted from *G. max* genome assembly version Glyma.Wm82.a2.v1 (https://soybase.org). To determine the gene annotations and Gene Ontology (GO) functions, extracted Glyma gene models were blasted against the TAIR 10 protein database (https://www.arabidopsis.org). Candidate genes were identified based on two methods: keyword searches and allele frequencies among extreme genotypes.

To identify candidate genes based on allele frequencies among extreme genotypes, 20 genotypes consisting of two groups of 10 genotypes from each phenotypic tail were selected for a given trait in each environment. Then, all SNPs within the LD region of a significant locus were isolated for the designated set of 20 genotypes. In the next step, the reference and alternate alleles among the two extreme groups of genotypes were identified, and the allele frequencies of an SNP for each group were calculated. The SNPs for which the reference allele frequency in one group was ≥ 80%, and the alternate allele frequency in the contrasting group was ≥ 80% were selected for candidate gene searches. Selected SNPs with these contrasting allele combinations were pursued if they were located within a gene model.

For the candidate genes identified by the above methods, the literature was explored for further information. In addition, the predicted effect of polymorphism on the gene models identified by both methods mentioned above was determined using SnpEff (v4.0) (Cingolani et al., 2012). The SNPeff score was calculated for each gene model based on the weighted sum of three main categories of variants (SNPeff_score = HIGH × 20 + MODERATE × 10 + LOW × 1) (Cingolani et al., 2012; Lovell et al., 2021). The description of the variant categories, “HIGH,” “MODERATE,” and “LOW” can be found at https://pcingola.github.io/SnpEff/se_inputoutput/#effect-prediction-details.

## 3 Results

### 3.1 Phenotypic diversity, relationships, and heritabilities

ANOVA revealed highly significant (*P* < 0.0001) genotype and genotype x environment interaction effects for δ^13^C, [N], and C/N ratio. The environment effect was also significant for δ^13^C (*P* < 0.0001), [N] (*P* < 0.01), and C/N (*P* < 0.01). For δ^15^N, genotype and environment effects were highly significant (*P* < 0.0001 and *P* < 0.001), but the genotype x environment interaction effect was marginal (*P* = 0.08). The observed environment and genotype x environment interaction effects were consistent with the considerable differences in environmental conditions between the two years. Although mean daily temperatures from planting to sampling were similar in the two years (21.3°C vs. 22.2°C), temperatures during early growth were higher (18.6°C vs. 16.9°C), and those later in the season were lower (June: 23.1°C vs. 24.7°C; July: 22.3°C vs. 24.9°C) in the first than the second year (**Supplementary Table S2**). Likely of particular importance for the observed environment and genotype x environment interaction effects was the much lower precipitation from planting to sampling in 2014 (273 mm) than in 2016 (384 mm), which primarily resulted from the much lower precipitation in July (41 mm vs. 274 mm), and despite June precipitation being greater in the first than the second year (164 mm vs. 29 mm). Genotypic effects of the within-environment ANOVA were highly significant (*P* < 0.0001) for all traits in both environments except for δ^15^N in 2014, where the genotype effect was significant at *P* < 0.05 level (**Table 1**).

**Table 1:** Descriptive statistics and heritability of the traits in two environments, 2014 and 2016.

Traits	Env	Mean	Max	Min	CV (%)	ANOVA: genotype		*H* ^2^
						*F*-value	*P*	
δ^13^C	2014	−27.56	−25.73	−31.70	−1.06	11.89	< 0.001	0.94
	2016	−28.71	−27.67	−29.78	−1.15	4.97	< 0.001	0.81
δ^15^N	2014	6.19	10.39	1.88	34.51	1.37	< 0.05	0.35
	2016	4.53	8.29	1.69	34.82	1.76	< 0.001	0.42
[N]	2014	30.14	43.37	21.36	11.91	3.36	< 0.001	0.75
	2016	24.98	31.85	14.69	13.18	2.58	< 0.001	0.63
C/N	2014	14.31	20.18	10.11	10.52	5.47	< 0.001	0.85
	2016	17.44	29.48	12.40	14.76	2.94	< 0.001	0.67

**Figure 1:**
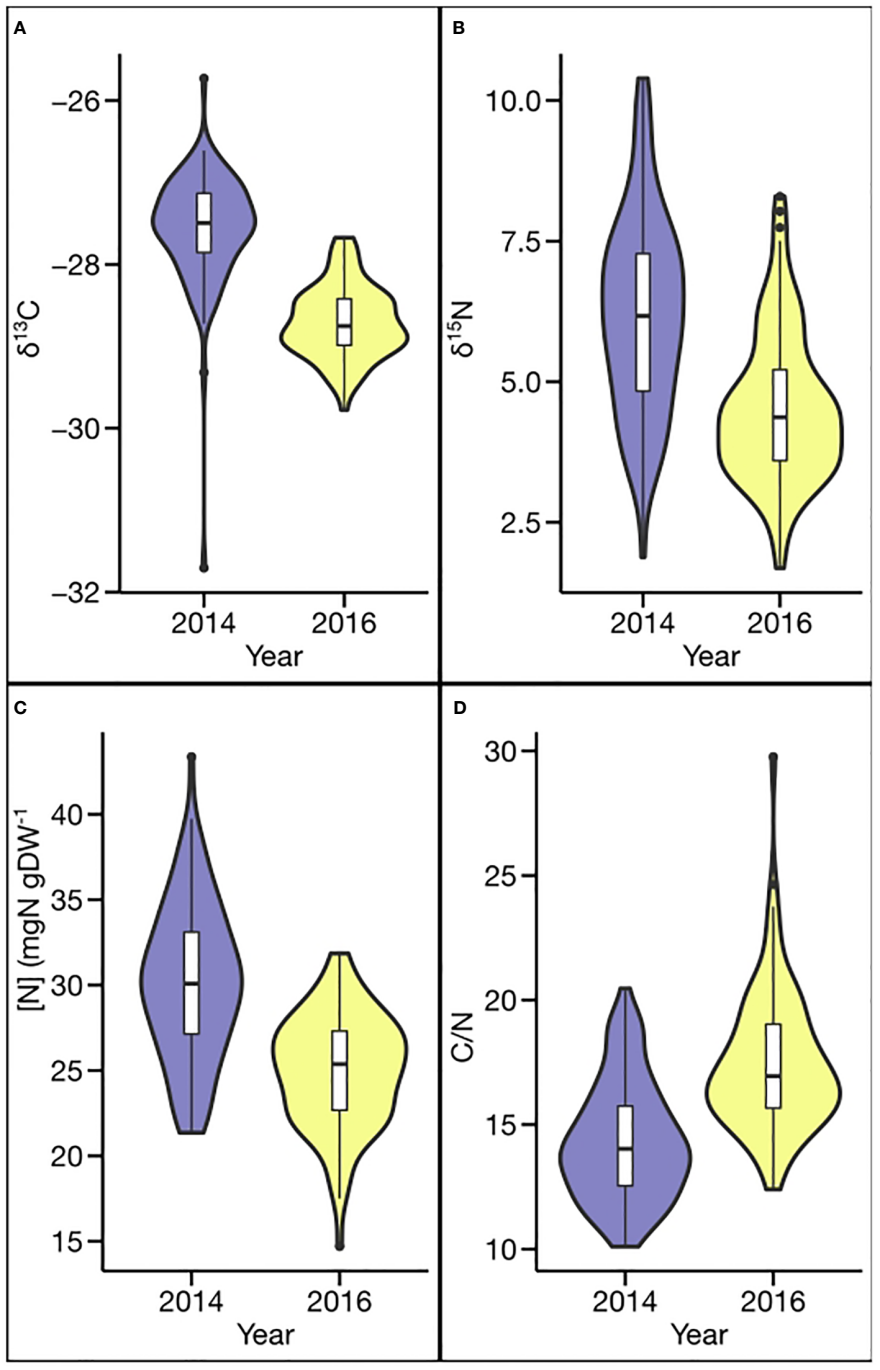
Phenotypic distribution for carbon- and nitrogen-related traits in different environments: **(A)** carbon isotope ratio (δ^13^C), **(B)** nitrogen isotope ratio (δ^15^N), **(C)** N concentration ([N]), and **(D)** carbon/nitrogen ratio (C/N).

Phenotypic values for all four traits exhibited a broad range in both environments, with δ^13^C ranging by 5.98 ‰ and 2.10 ‰, δ^15^N by 8.51‰ and 6.61 ‰, [N] by 22.01 mg g^−1^ and 17.16 mg g^−1^, and C/N by 10.36 and 17.37, in 2014 and 2016, respectively (**Table 1** and **Figure 1**). The greater ranges observed in 2014 for δ^13^C, δ^15^N, and [N] also were associated with greater mean values (δ^13^C −27.56 vs. −28.71 ‰; δ^15^N 6.19 vs. 4.53 ‰; and [N] 30.14 vs. 24.98 mg g−^1^). In contrast, the mean C/N ratio (14.31 vs. 17.44) and the range in C/N ratio (10.36 vs. 17.37) were smaller in 2014 than in 2016.

The considerable differences in environmental conditions were also reflected in the relatively weak correlations of phenotypic data between environments, namely, *r* = 0.29 (*P* < 0.01) for δ^13^C, *r* = 0.20 (*P* < 0.06) for δ^15^N, *r* = 0.28 (*P* < 0.01) for [N], and *r* = 0.32 (*P* < 0.01) for C/N ratio (**Table 2**). Not surprisingly, strong negative correlations between the C/N ratio and [N] were observed in each environment and across both years (*r* = −0.93 or −0.97; *P* < 0.001). In contrast, relationships were not consistent among other traits when analyzed by environment, but, when analyzed across environments, the C/N ratio and δ^13^C (*r* = −0.21; *P* < 0.05) as well as C/N ratio and δ^15^N (*r* = −0.23; *P* < 0.05) were negatively correlated, and δ^13^C and δ^15^N (*r* = 0.21; *P* < 0.05) were positively correlated.

**Table 2:** Pearson correlation coefficients between the traits in two environments and across the environments (AEs).

	δ^13^C_14	δ^15^N_14	[N]_14	C/N_14	δ^13^C_16	δ^15^N_16	[N]_16	C/N_16	δ^13^C-AE	δ^15^N-AE	[N]-AE	C/N-AE
**δ^13^C_14**	1.00											
**δ^15^N_14**	−0.10	1.00										
**[N]_14**	−0.18	0.39***	1.00									
**C/N_14**	0.17	−0.33***	−0.97***	1.00								
**δ^13^C_16**	0.29**	0.14	0.27**	−0.32**	1.00							
**δ^15^N_16**	0.01	0.20	−0.02	−0.02	0.45***	1.00						
**[N]_16**	0.01	−0.04	0.28**	−0.32**	0.25*	0.00	1.00					
**C/N_16**	−0.03	0.00	−0.28**	−0.32**	−0.24*	−0.06	−0.93***	1.00				
**δ^13^C-AE**	0.71***	−0.01	0.02	−0.05	0.79***	0.32**	0.12	−0.16	1.00			
**δ^15^N-AE**	−0.09	0.76***	0.29**	−0.34***	0.38***	0.73***	−0.01	−0.07	0.21*	1.00		
**[N]-AE**	−0.18	0.19	0.80***	−0.81***	0.34***	−0.01	0.75***	−0.68***	0.15	0.20	1.00	
**C/N-AE**	0.09	−0.17	−0.66***	0.72***	−0.35***	−0.07	−0.79***	0.84***	−0.21*	−0.23*	−0.93***	1.00

*P < 0.05, **P < 0.01, and ***P < 0.001.

Broad sense heritability estimates differed substantially between the traits and, to a lesser extent, between the two environments for a given trait. Heritability estimates for 2014 and 2016 were greatest for δ^13^C (0.94 and 0.81), lowest for δ^15^N (0.35 and 0.42), and intermediate for C/N ratio (0.85 and 0.67) and [N] (0.75 and 0.63). When combined across the two years, heritabilities were 0.58, 0.24, 0.39, and 0.44 for δ^13^C, δ^15^N, [N], and C/N ratio, respectively (**Table 1**).

### 3.2 SNP marker distribution and population structure

**Figure 2:**
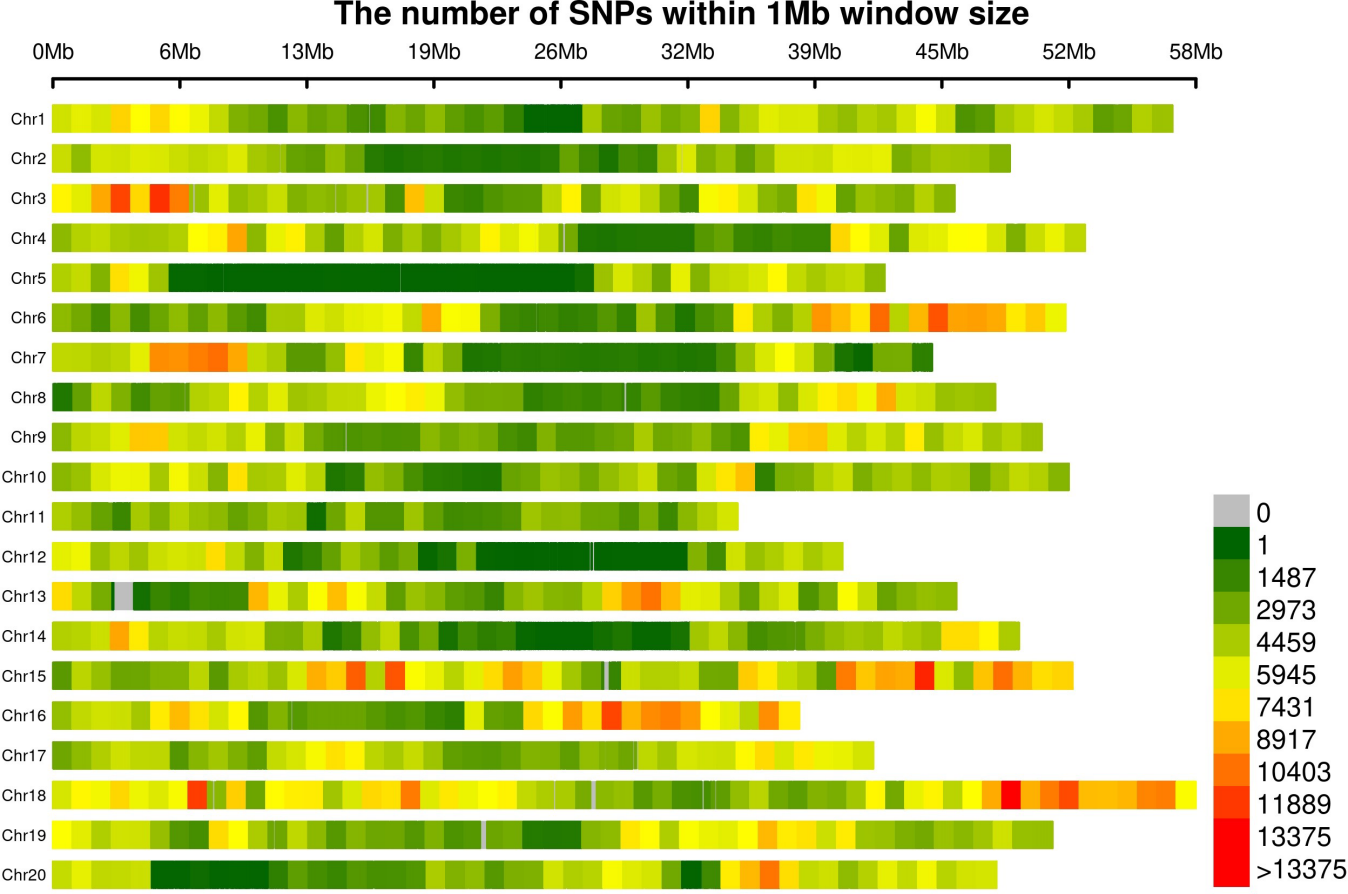
Marker density plot within 1 MB region across the whole genome.

The 4,108,002 SNP markers retained after removing monomorphic markers, markers with 50% missing data, and markers with less than 5% minor allele frequency were widely distributed across the genome (**Figure 2**). The average marker density was 4,283 SNPs Mbp^−1^, with the highest marker density on Gm18 (6,234 SNPs Mbp^−1^) and the lowest marker density on Gm05 (2,544 SNPs Mbp^−1^) (**Supplementary Table S3**). Within chromosomes, the distinct, typical pattern in SNP densities largely aligned with heterochromatic and euchromatic regions, with euchromatic regions on several chromosomes exceeding densities of 10K SNPs Mbp^−1^ (**Figure 2**). The average marker density in the euchromatic regions was 5690 SNPs Mbp^−1^, compared to 3110 SNPs Mbp^−1^ in the heterochromatic regions (**Supplementary Table S3**).

**Figure 3:**
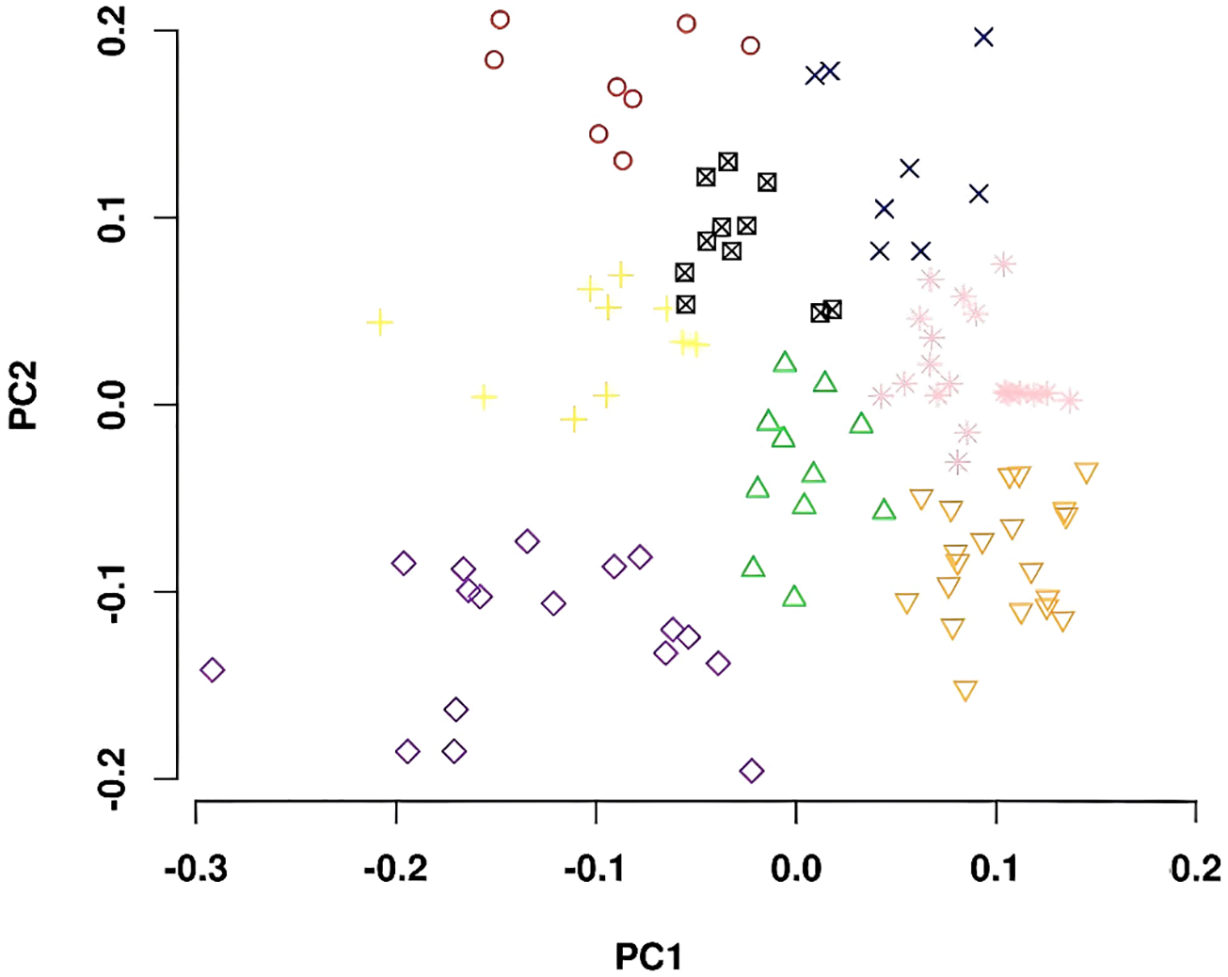
Principal component analyses plots showing the first two principal components separated the whole germplasm panel into eight subpopulations.

The genotypes comprising the diversity panel were divided into eight subgroups based on principle component analysis (**Figure 3**). The subpopulations were not associated with any specific MG or location of origin.

### 3.3 Marker-trait associations

**Figure 4:**
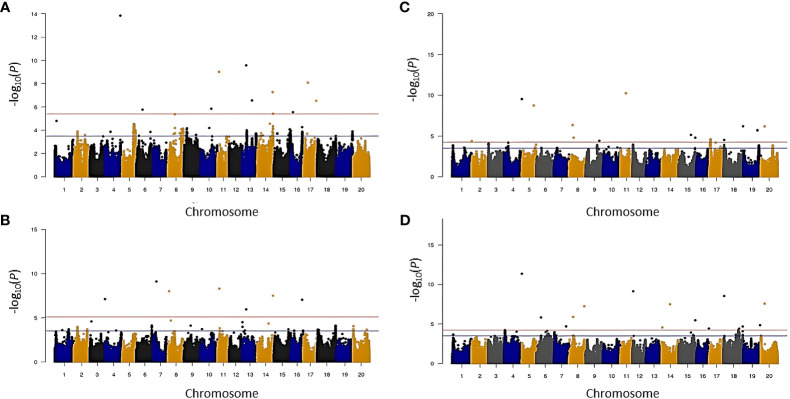
Manhattan plot showing significant marker-trait association for carbon isotope ratio (δ^13^C) and nitrogen isotope ratio (δ^15^N) in 2014 and 2016 environment before stepwise regression. **(A)** δ^13^C in 2014, **(B)** δ^13^C in 2016, **(C)** δ^15^N in 2014, and **(D)** δ^15^N in 2016.

**Figure 5:**
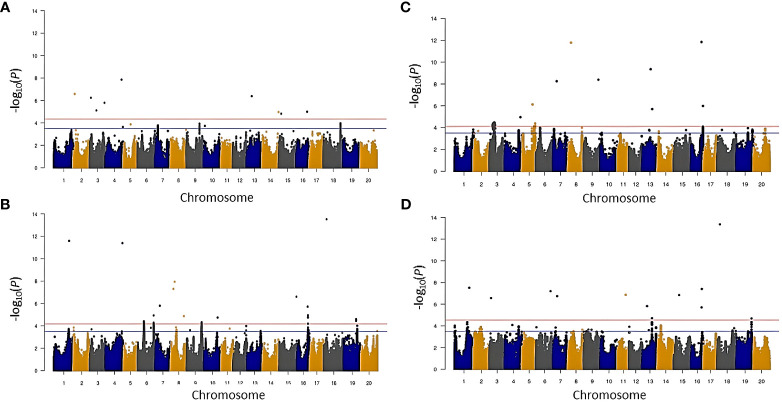
Manhattan plot showing significant marker-trait association for N concentration ([N]) and carbon/nitrogen ratio (C/N) in 2014 and 2016 environment before stepwise regression. **(A)** ([N]) in 2014, **(B)** ([N]) in 2016, **(C)** C/N in 2014, and **(D)** C/N in 2016.

Given the significant differences between the two years, marker associations were identified based on BLUP values calculated for each trait for each year using the FarmCPU model (**Figures 4**, **5**). To declare a marker-trait association significant, markers had to either pass (1) a -log (*p*) value threshold determined by the FarmCPU model [-log (*p*) values ranged from 4.11 to 5.40; **Supplementary Table S4**], (2) a -log (*p*) value threshold of 3.5 in both environments or (3) a -log (*p*) value threshold of 3.5 in one of the two environments while also being within 1.5 Mbp of a previously identified locus documented in the literature. The SNPs identified in this manner were subjected to a stepwise regression to account for marker × marker interactions and retain only major and independent markers. The resulting SNPs and putative loci identified for each trait are listed in **Table 3** and **Supplementary Table S6**.

**Table 3:** Significant markers and loci associated with carbon istope ratio (δ13C), nitrogen isotope ratio (δ15N), N content and C/N ratio in two environments, 2014 and 2016, after stepwise regression.

Loci	SNP	-log_10_ (p)	Effect	Env	Previous literature	Candidate gene, Glyma model	Araba Gene Model_Annotation_GO Biological Process
δ^13^C							
1	Chr01_4685420	4.79	−0.21	2014		Glyma.01G042100	AT1G69850.1_Nitrate Transporter 1:2_Regulation of stomatal movement
						Glyma.01G042900	AT4G35540.1_Zinc ion binding;transcription regulators_Stomatal lineage progression_Hypothetical protein_Stomatal lineage progression
2	Chr02_11005534	3.76	−0.11	2016	δ^13^C^AD^	Glyma.02G113800	AT2G03420.1_Hypothetical protein_Stomatal complex morphogenesis
	Chr02_11008264	3.80	−0.11	2016			
	Chr02_11011600	3.97	−0.11	2016			
3	Chr04_118837	7.12	0.17	2016		Glyma.04G001500	AT4G35250.1_High Chlorophyll Fluorescence Phenotype 244_Photosynthesis, Light reaction; Photosystem II assembly
						Glyma.04g001000**^φ^**	NA_NA_NA
4	Chr07_9212793	9.11	0.32	2016		Glyma.07G100200	AT1G73690.1_Cyclin-dependent kinase D1;1 _Stomatal lineage progression
						Glyma.07G102300	AT4G30720.1_Pigment Defective 327_Photosynthesis
5	Chr08_3203043	8.01	−0.20	2016		Glyma.08G037300	AT4G28980.2_CDK-activating kinase 1AT_Stomatal lineage progression
						Glyma.08G040500	AT1G49040.1_Stomatal Cytokinesis Defective 1_Guard mother cell cytokinesis
						Glyma.08g042600**^φ^**	AT2G01660.1_plasmodesmata-located protein 6_NA
6	Chr10_4512696	3.72	0.12	2016	δ^13^C^E^		
7*	Chr13_11599577	9.57, 5.96	−0.34	2014, 2016		Glyma.13G028200	ATCG00020.1_Photosystem II Reaction Center Protein A_Photosynthesis; Photosynthesis, Light reaction; Photosynthetic electron transport in photosystem II; Photosystem II assembly
						Glyma.13G030300	AT3G45140.1_Lipoxygenase 2_Response to water deprivation
						Glyma.13G030900	AT1G01720.1_NAC domain Transcriptional regulator superfamily protein ATAF1_Response to water deprivation
						Glyma.13G031500	AT1G44110.1_Cyclin A1;1_Stomatal complex morphogenesis
						Glyma.13G039700	AT5G48940.1_RGF1 Insensitive 2_Stomatal complex morphogenesis
						Glyma.13G040100	AT5G53210.1_Speechless_Stomatal complex development; Stomatal complex morphogenesis
						Glyma.13G042800	AT1G60600.2Aberrant Chloroplast Development 4_Stomatal complex morphogenesis
8	Chr14_45828579	7.27	−0.15	2014	δ^13^C^E^		
9	Chr14_46907247	7.50	−0.13	2016		Glyma.14G202700	AT5G07070.1_CBL-interacting protein kinase 2_Stomatal movement
						Glyma.14G202900	AT2G30370.1_Epidermis Patterning Factor (EPF1)-like 6/CHALLAHNegative regulation of stomatal complex development
						Glyma.14G203000	AT2G30360.1_CBL-Interacting Protein Kinase 11Abscisic acid mediated signaling pathway; Stomatal movement
						Glyma.14G203100	AT2G30370.1_Epidermis Patterning Factor (EPF1)-like 6/CHALLAHNegative regulation of stomatal complex development
						Glyma.14G203700	AT5G58350.1_With no lysine (K) kinase 4_Cellular response to water deprivation
10	Chr16_6176703	5.54	0.19	2014	δ^13^C^E^	Glyma.16G064700	AT1G60600.1_Aberrant Chloroplast Development 4_Stomatal complex morphogenesis
11	Chr17_13486270	8.08	0.23	2014	δ^13CD^		
δ^15^N							
1	Chr01_2219735	3.59	−0.37	2014	δ^15^N^E^		
	Chr01_2220137	3.82	−0.35	2014			
2	Chr02_180074	4.37	0.25	2014			
3*	Chr04_52111357	9.53, 11.35	0.54	2014, 2016			
4	Chr05_34389904	8.75	−0.38	2014		Glyma.05G149500	AT4G34880.1_Amidase family proteinAcrylonitrile catabolic process; Aldoxime metabolic process
5	Chr06_13425562	5.82	0.27	2016			
6	Chr07_35287527	4.69	0.37	2016		Glyma.04g225600**^φ^**	
7	Chr08_9703532	6.34	0.28	2014			AT2G38290.1_Hypothetical Protein 30-2, Trna Import Component 2 Mitochondrion organization, tRNA import into mitochondrion
8	Chr08_11251742	5.89	−0.23	2016		Glyma.08G152700	AT5G26880.2_AGAMOUS-like 26_RNA processing; Cellular response to nitrogen starvation; Terpenoid biosynthetic process
9	Chr11_15599785	10.25	0.39	2014			
10	Chr12_2123301	9.13	−0.34	2016			
11	Chr14_1119646	4.56	−0.41	2016			
12	Chr15_35597445	5.14	0.39	2014			
13*	Chr15_48318525	4.79, 5.47	0.39	2014, 2016	δ^15^N^E^		
14	Chr16_36341221	4.42	0.17	2016			
15	Chr17_3444556	4.39	−0.30	2014			
	Chr17_3456184	4.56	−0.31	2014			
16	Chr18_1416686	3.59	−0.2	2014			
17*	Chr18_1557539	4.51, 8.53	0.19	2014, 2016			
18	Chr18_56639347	6.19	−0.37	2014			
19	Chr19_40435224	5.69	−0.30	2014			
20	Chr19_44730876	3.72	0.28	2014	δ^15^N^E^		
	Chr19_44733269	3.84	0.28	2014			
21	Chr19_48192901	4.84	−0.20	2016		Glyma.19G227100	AT1G09270.2_Importin alpha isoform 4_host response to induction by symbiont of tumor, nodule or growth in host
						Glyma.19G227200	AT1G09270.1_Importin alpha isoform 4_host response to induction by symbiont of tumor, nodule or growth in host
22*	Chr20_10846267	6.18, 7.57	0.59	2014, 2016			
[N]							
1	Chr01_44121372	11.59	0.93	2016			
2	Chr02_4085484	6.58	−0.94	2014		Glyma.02G043700	AT2G38290.1_Ammonium transporter 2_Cellular response to nitrogen starvation;
3	Chr03_3035296	6.24	0.87	2014	Ureide^CE^	Glyma.03G028000	AT4G08870.1_Arginine Amidohydrolase 2_Ornithine metabolic process; Polyamine metabolic process; Proline metabolic process; Putrescine metabolic process; Tyrosine metabolic process
						Glyma.03G028100	AT1G02020.1_Nitroreductase family protein_Metabolic process
4	Chr03_19093359	5.11	1.02	2014			
5	Chr03_42892475	5.79	1.48	2014			
6	Chr04_47257970	7.85	−1.86	2014			
7	Chr04_49554285	11.39	−0.90	2016		Glyma.04g225600**^φ^**	AT2G38290.1_Hypothetical Protein 30-2, Trna Import Component 2_Mitochondrion organization, tRNA import into mitochondrion
8	Chr06_17797975	4.23	−0.61	2016			
	Chr06_17898717	4.33	−0.63	2016			
9	Chr06_18389101	4.22	−0.56	2016	Ureide^C^	Glyma.06G213100	AT2G01570.1_Repressor Of GA1-3 1_Regulation of nitrogen utilization;
10	Chr06_46685765	3.81	−0.54	2016			
	Chr06_46690437	3.73	−0.54	2016			
	Chr06_46693667	3.90	−0.55	2016			
	Chr06_46699466	3.56	−0.52	2016			
	Chr06_46703572	4.02	−0.54	2016			
	Chr06_46703908	4.05	−0.55	2016			
	Chr06_46717791	3.86	−0.53	2016			
	Chr06_46718629	3.84	−0.53	2016			
	Chr06_46721520	4.21	−0.56	2016			
	Chr06_46727008	3.73	−0.53	2016			
	Chr06_46748617	3.97	−0.56	2016			
	Chr06_46748622	3.74	−0.54	2016			
11	Chr07_13483940	5.79	0.60	2016	Ureide^C^		
12	Chr08_8526365	7.30	0.75	2016			
13	Chr08_12245091	7.94	−1.53	2016			
14	Chr08_39254642	4.87	0.61	2016			
15	Chr09_43775692	4.22	−0.62	2016			
16	Chr10_40392511	4.73	0.51	2016	Ureide^C^		
17	Chr14_48265528	4.97	0.89	2014	[N]^B^; Ureide^C^		
18	Chr15_50601295	6.60	0.56	2016			
19*	Chr16_31477020	4.99	−0.89	2014	[N]^E^	*Glyma.16G156700*	AT3G54220.1_SCARECROW, Shoot Gravitropism 1_Regulation of nitrogen utilization;
						*Glyma.16g154100* **^+φ^**	AT5G19790.1_Related To AP2 11_Cellular response to potassium ion; Post-embryonic root development; Response to ethylene stimulus; Response to reactive oxygen species
						*Glyma.16g154700* **^+φ^**	AT5G19500.1_ Tryptophan/tyrosine permease_Amino acid transport; Purine nucleobase transport
						Glyma.16g154800**^φ^**	AT5G14990.1_WPP domain associated protein_Biological process
						*Glyma.16g155000* **^+φ^**	AT2G37170.1_plasma membrane intrinsic protein 2/Aquaporin Transporter_Response to abscisic acid stimulus; Response to salt stress; Response to water deprivation; Transmembrane transport; Water transport
						Glyma.16g155100**^φ^**	AT2G37170.1_plasma membrane intrinsic protein 2/Aquaporin Transporter_Response to abscisic acid stimulus; Response to salt stress; Response to water deprivation; Transmembrane transport; Water transport
						Glyma.16g155200**^φ^**	AT2G28320.1_ Pleckstrin homology (PH) and lipid-binding START domains-containing protein)_Signal transduction
						*Glyma.16g155300* **^+φ^**	AT3G60530.1_GATA transcription factor 4_Circadian rhythm; Positive regulation of transcription, DNA-dependent; Regulation of transcription, DNA-dependent; Response to light stimulus
						*Glyma.16g155400* **^φ^**	AT2G39000.1_Acyl-CoA N-acyltransferases (NAT) superfamily protein)_Metabolic process
						*Glyma.16g155500* **^φ^**	AT2G28370.1_(Uncharacterized protein family (UPF0497))/Casp-Like Protein 5A2, CASPL5A2_Golgi vesicle transport; Biological process; Cell growth; Cell morphogenesis; Phosphatidylinositol biosynthetic process
						*Glyma.16g155600* **^+φ^**	AT5G13570.1_Decapping 2_Cellular macromolecule catabolic process; Deadenylation-independent decapping of nuclear-transcribed mRNA; Fatty acid beta-oxidation; mRNA catabolic process; Nuclear-transcribed mRNA catabolic process; Post-embryonic development; Posttranscriptional gene silencing; Primary shoot apical meristem specification; Protein import into peroxisome matrix
						*Glyma.16g155700* **^+φ^**	AT3G24170.1_Glutathione-disulfide reductaseCell redox homeostasis; Glutathione metabolic process; Oxidation-reduction process; Toxin catabolic process
						Glyma.16g156400**^φ^**	AT1G34370.1_(C2H2 and C2HC zinc fingers superfamily protein)_Regulation of transcription, DNA-dependent; Response to acidity; Response to aluminum ion
	Chr16_31554412	5.71	−0.67	2016			
20	Chr18_7624586	13.52	1.53	2016			
21	Chr19_36299796	4.45	−0.85	2016			
	Chr19_36432092	4.59	−0.85	2016			
C/N							
1	Chr01_44150278	7.51	−0.47	2016		Glyma.01G129000	AT5G01750.2Protein of unknown function (DUF567)_Cellular response to nitrogen starvation;
2	Chr03_4533967	6.56	−0.54	2016		Glyma.01G129100	AT5G01750.2Protein of unknown function (DUF567)_Cellular response to nitrogen starvation;
3	Chr03_14622737	4.31	0.38	2014			
4	Chr03_15071708	4.28	0.38	2014			
5*	Chr04_41951439	3.68	0.37	2014			
	Chr04_41997069	3.56	0.37	2016			
	Chr04_42002228	3.51	0.36	2016			
	Chr04_42002635	3.91	0.39	2016			
	Chr04_42002928	3.57	0.37	2016			
	Chr04_42003014	3.52	0.36	2016			
	Chr04_42004874	3.52	0.37	2016			
	Chr04_42172924	3.76	0.41	2014			
6	Chr04_47257970	4.95	0.64	2014			
7	Chr05_39258552	4.39	−0.6	2014			
8	Chr06_43407492	7.20	−0.56	2016			
9*	Chr07_10553068	8.25	0.72	2014			
	Chr07_11960539	6.74	−0.58	2016		Glyma.07G114200	AT1G02205.2ECERIFERUM 1_Aldehyde catabolic process; Alkane biosynthetic process; Cuticle development; Response to water deprivation; Wax biosynthetic process
10	Chr08_9154847	11.78	0.79	2014		Glyma.08G118500**^ψ^**	AT1G29290.1_C-Terminally Encoded Peptide 14_Cellular response to nitrogen starvation, Nitrate import, Regulation of root development, Response to carbon dioxide
11	Chr09_43743962	8.39	0.67	2014			
12	Chr11_24349199	6.86	1.24	2016		Glyma.11G169700	AT1G07110.1_Fructose-2,6-bisphosphatase_Regulation of carbon utilization
						Glyma.11G172000	AT1G07110.1_Fructose-2,6-bisphosphatase_Regulation of carbon utilization
						Glyma.11G176800	AT3G54140.1_Peptide transporter 1_Nitrogen compound metabolic process
13	Chr13_13907234	5.82	0.72	2016		Glyma.13G046200	AT5G38410.1_Rubisco Small Subunit 3b_Carbon fixation; Photosynthesis
						Glyma.13g045900**^φ^**	AT5G38380.1_ zinc transporter_Biological process
						Glyma.13g046300**^φ^**	AT3G02550.1_LOB domain-containing protein 41_Regulation of hydrogen peroxide metabolic process; Regulation of transcription, DNA-dependent; Response to hypoxia; Systemic acquired resistance, Salicylic acid mediated signaling pathway
14	Chr13_24887452	9.35	0.67	2014			
15	Chr13_28993838	4.70	0.41	2016			
16	Chr13_29565172	5.70	−0.48	2014		Glyma.13g181500**^φ^**	AT2G20760.1_Clathrin Light 62 Chain1_clathrin-dependent endocytosis
17	Chr14_9986941	3.53	0.35	2016			
18	Chr15_15867944	6.84	1.04	2016			
19*	Chr16_31477020	11.84	0.80	2014		*Glyma.16G156700*	AT3G54220.1_Scarecrow, Shoot Gravitropism 1_Regulation of nitrogen utilization
						*Glyma.16g154100* **^φ^**	AT5G19790.1_Related To AP2 11_Cellular response to potassium ion; Post-embryonic root development; Response to ethylene stimulus; Response to reactive oxygen species
						*Glyma.16g154700* **^+φ^**	AT5G19500.1_ Tryptophan/tyrosine permease_Amino acid transport; Purine nucleobase transport
						*Glyma.16g155000* **^+φ^**	AT2G37170.1_plasma membrane intrinsic protein 2/Aquaporin Transporter_Response to abscisic acid stimulus; Response to salt stress; Response to water deprivation; Transmembrane transport; Water transport
						*Glyma.16g155200* **^φ^**	AT2G28320.1_ Pleckstrin homology (PH) and lipid-binding START domains-containing protein)_Signal transduction
						*Glyma.16g155300* **^+φ^**	AT3G60530.1_GATA transcription factor 4_Circadian rhythm; Positive regulation of transcription, DNA-dependent; Regulation of transcription, DNA-dependent; Response to light stimulus
						*Glyma.16g155400* **^φ^**	AT2G39000.1_Acyl-CoA N-acyltransferases (NAT) superfamily protein)_Metabolic process
						*Glyma.16g155500* **^φ^**	AT2G28370.1_(Uncharacterized protein family (UPF0497))/Casp-Like Protein 5A2, CASPL5A2_Golgi vesicle transport; Biological process; Cell growth; Cell morphogenesis; Phosphatidylinositol biosynthetic process
						*Glyma.16g155600* **^+φ^**	AT5G13570.1_Decapping 2_Cellular macromolecule catabolic process; Deadenylation-independent decapping of nuclear-transcribed mRNA; Fatty acid beta-oxidation; mRNA catabolic process; Nuclear-transcribed mRNA catabolic process; Post-embryonic development; Posttranscriptional gene silencing; Primary shoot apical meristem specification; Protein import into peroxisome matrix
						*Glyma.16g155700* **^+φ^**	AT3G24170.1_Glutathione-disulfide reductase_Cell redox homeostasis; Glutathione metabolic process; Oxidation-reduction process; Toxin catabolic process
	Chr16_31653264	5.70	0.54	2016			
20	Chr16_32580938	7.40	1.11	2016			
21	Chr18_7624586	13.37	−1.10	2016			
22	Chr19_44735470	4.68	−0.48	2016			

Bold * Loci that were detected in both years.

^φ^Candidate genes based on allelic combination.

^ψ^Candidate gene identified by both “keyword search” and “allelic combination.

^+^Significant allele combination score in both environment.

Italic candidate genes: candidate genes identified for multiple traits.

^A^ Dhanapal et al., 2015a; ^B^ Dhanapal et al., 2015b; ^C^ Ray et al., 2015, ^D^ Kaler et al., 2017; ^E^ Steketee et al., 2019.

Thirteen SNPs marking 11 putative loci were associated with δ^13^C (**Table 3** and **Supplementary Table S6**). A single locus each was identified on Gm01, Gm02, Gm04, Gm07, Gm08, Gm10, Gm13, Gm16, and Gm17, and two loci were identified on Gm14. Among these loci, the one on Gm13 was identified in both years. Except for the locus on Gm02 marked by three SNPs, all loci were identified based on a single SNP.

For δ^15^N, 25 SNPs tagged 22 putative loci distributed across all chromosomes except Gm03, Gm09, Gm10, and Gm13 (**Table 3** and **Supplementary Table S6**). Of the 22 putative loci, four (loci 3, 13, 17, and 22) were detected in both environments, whereas the other 18 loci were identified only in one of the two environments. Nineteen loci were marked by single SNPs, and three loci (1, 15, and 20) on Gm01, Gm17, and Gm19, respectively, were marked by two SNPs each.

Among the four traits, the largest number of SNPs, 35, was identified for [N] (**Table 3** and **Supplementary Table S6**). These 35 SNPs tagged 21 putative loci, one of which, locus 10 on Gm06, was marked by 12 significant SNPs, and two others, locus 8 on Gm06 and locus 19 on Gm16, were marked by two SNPs. The 21 loci were located on 14 different chromosomes, with more than one locus identified on Gm03 (three loci), Gm04 (two loci), Gm06 (three loci), and Gm08 (three loci). One of the 21 loci, namely, locus 19 on Gm16, was detected in both environments.

For C/N ratio, 22 putative loci were tagged by 31 SNPs (**Table 3** and **Supplementary Table S6**). The 22 putative loci were located on 15 chromosomes, including two loci on Gm04 and Gm16, three on Gm03, and four on Gm13. One of the loci, locus 5 on Gm04, was anchored by eight SNPs and was identified in both environments. Locus 9 on Gm07 and locus 19 on Gm16 were also identified in both environments and were marked by two SNPs each.

After the stepwise regression procedure, local LD was estimated for each significant marker retained. The span of the LD regions for significant markers varied greatly, ranging from 4.4 Kb (marker Chr13_28993838 associated with C/N) to 7,427.2 Kb (marker Chr13_11599577 associated with δ^13^C) (**Supplementary Table S5**). Across all markers, the average span of the LD regions was 802.06 Kb.

### 3.4 Candidate genes

All gene models within the LD regions of the significant markers were extracted and blasted against the *Arabidopsis* protein database to obtain the annotations and GO functions of the genes. Thirty-nine candidate genes were identified based on keyword searches executed for the gene annotations and GO functions, and 20 were identified based on the allelic combination analysis of 10 genotypes from each of the phenotypic tails (**Table 3**; **Supplementary Tables S7**, **S8**).

Based on keyword searches, 21 candidate genes were identified within the LD regions of the markers associated with δ^13^C. Among these, two gene models encode for transcription factors, namely, Glyma.13G030900 encoding *No Apical Meristem (NAC)* domain transcriptional regulator superfamily protein (ANAC002/ATAF1) and Glyma.13G040100 encoding *Speechless (SPCH)*. Some notable candidate genes are Glyma.01G042100, encoding *Nitrate Transporter 1:2 (NRT1:2)*, Glyma.08G040500, encoding *Stomatal Cytokinesis Defective 1* (*SCD1*), Glyma.13G031500 encoding *Cyclin A1;1 (CYCA1;1)*, Glyma.14G202700 encoding *CBL-interacting protein kinase 2 (CIPK2, SnRK3.2)* and Glyma.14G202900 encoding *EPIDERMIS PATTERNING FACTOR (EPF1)-LIKE 6* or *CHALLAH (CHAL, EPFL6)* (**Table 3** and **Supplementary Table S7**). Two candidate genes, Glyma.04g001000 (locus 3, no homologue in Arabidopsis) and Glyma.08g042600 (locus 5) encoding a *Plasmodesmata-located protein 6* (*PDLP6*) were identified for δ^13^C based on the allelic combination analysis, but no-GO annotations were available for either candidate gene (**Table 3** and **Supplementary Table S8**).

Four candidate genes were identified for δ^15^N based on the keyword searches of the gene annotation and GO functions of all genes within the LD regions of the δ^15^N associated loci. These are Glyma.05G149500 (locus 4) encoding an Amidase family protein, Glyma. 08G152700 (locus 8) encoding *AGAMOUS-like 26 (AGL26)*, and Glyma.19G227100 and Glyma.19G227200 (both locus 21) both encoding *Importin alpha isoform 4 (IMPA-4)* (**Table 3** and **Supplementary Table S7**). The Glyma.05G149500 gene at locus 4 is only 20 Kb downstream of the significant marker anchoring the locus. One candidate gene, Glyma.08g124800 at locus 7, was identified for δ^15^N based on allelic combination analysis. This gene is annotated as Protein kinase superfamily protein but was not linked to any nitrogen fixation related function based on GO annotation (**Table 3** and **Supplementary Table S8**).

For [N], five candidate genes were identified based on the keyword searches executed on gene annotations and GO functions. These are Glyma.02G043700 (locus 2) encoding *Ammonium transporter 2* (*AMT2*), Glyma.03G028000 (locus 3) encoding *ARGININE AMIDOHYDROLASE 2* (*ARGAH2*), Glyma.03G028100 (locus 3) encoding a Nitroreductase family protein, Glyma.06G213100 (locus 9) encoding *REPRESSOR OF GA1-3 1* (*RGA/RGA1*), and Glyma.16G156700 (locus 19) encoding *SCARECROW/SHOOT GRAVITROPISM* (*SCR/SGR1*) (**Table 3** and **Supplementary Table S7**). Glyma.02G043700 on locus 2 and Glyma.03G028000 on locus 3 are located 14.64 Kb downstream and 34.79 Kb upstream of the respective significant markers. Based on the examination of allelic combinations, 13 candidate genes were identified for [N], including 12 that were located at locus 19 and one at locus 7 (**Table 3** and **Supplementary Table S8**). Of these 13 genes, two were annotated as transcription factors, namely RELATED TO AP2 11 (RAP2.11) and GATA transcription factor 4 (GATA4); however, none of the 13 candidate genes identified based on allelic combination were overlapping with the candidate genes identified based on keyword searches.

For the C/N trait, eight candidate genes were identified based on the keyword searches on the gene annotations and GO functions. A gene encoding a protein of unknown function (Glyma.01G12900 and Glyma.01G129100) was identified at locus 1. Glyma.07G114200 (locus 9) encoding *ECERIFERUM 1 (CER1)* and Glyma.16G156700 (locus 19) encoding *SCARECROW* (*SCR*) or *SHOOT GRAVITROPISM 1* (*SGR1*) were detected at 34.87 Kb downstream and 28.14 Kb upstream of the significant markers, respectively. In addition to these, three genes, Glyma.11G169700 and Glyma.11G172000 both encoding *Fructose-2,6-bisphosphatase* (*FKFBP*) and Glyma.11G176800 encoding *Peptide transporter 1* (*PTR1*), were detected on locus 12 (**Table 3** and **Supplementary Table S7**). In addition, a total of 13 candidate genes were identified for C/N based on the allelic combination analysis (**Table 3** and **Supplementary Table S8**). Nine of these candidate genes were found at locus 19 on Gm16 and also were associated with [N] (locus 19), including the two transcription factors RAP2.11 and GATA4. The other four candidate genes were located on Gm08 and Gm13 and were associated with loci 10, 13, or 16. One candidate gene Glyma.08g118500 (locus 10) for C/N was detected based on the keyword searches and based on allelic combination. This gene encodes a *C-TERMINALLY ENCODED PEPTIDE 14* and has a GO annotation indicating a role in N dynamics.

## 4 Discussion

### 4.1 Phenotypes

Genotypic variation was significant for all traits (δ^13^C, [N], δ^15^N, and C/N) in both years (**Table 1** and **Figure 1**). The genotypic variation in δ^13^C is consistent with previous studies, which also documented significant genotypic variation. The estimated heritabilities for δ^13^C of 0.94 and 0.81 in 2 years are relatively higher compared to the heritability estimates (0.59–0.72) for δ^13^C in diversity panels consisting primarily of MGIV or MGVI-MGVIII soybean from the USDA soybean germplasm collection (Dhanapal et al., 2015a; Kaler et al., 2017; Steketee et al., 2019). Heritability estimates for [N] were moderate to high (0.75 and 0.63) and in agreement with those reported previously, which ranged from 0.37 to 0.73 (Dhanapal et al., 2015b; Steketee et al., 2019). For C/N, heritability estimates of 0.85 and 0.67 in the current study also exceeded the range of 0.37–0.59 reported previously by (Dhanapal et al., 2015b). The lowest heritabilities among the four traits were estimated for δ^15^N (0.35 and 0.42). The comparatively low heritabilities are consistent with Steketee et al. (2019) and Bazzer et al. (2020c), who also reported low to moderate heritability for δ^15^N ranging from 0.07 to 0.40 in a GWAS and biparental mapping study, respectively. Low heritability for N_2_ fixation was also documented by Dhanapal et al. (2015b) and Ray et al. (2015) based on N derived from the atmosphere (Ndfa) (0.15–0.26) and shoot ureide concentration (0.23–0.38), respectively. Low to moderate heritability for δ^15^N in both years in the current study indicates considerable environmental influence on this trait, possibly due to spatial within-field variability in soil mineral N concentration (Steketee et al., 2019), which is well known to influence SNF in soybean (Evans, 2001; Donahue et al., 2020; Moro Rosso et al., 2023). Nonetheless, significant genotypic variation and heritability estimates indicate potential for genetic improvement for all four traits.

Relationships among the different traits generally varied between the 2 years, except for strong negative correlations (−0.97, *p* < 0.0001) and (−0.93, *p* < 0.0001) between shoot C/N ratio and shoot [N], which were expected (**Table 2**). These results suggest that environmental factors prevailing in the 2 years influenced traits to different extents. Few studies have compared the relationship between δ^13^C and δ^15^N or the other traits examined here in soybean. Steketee et al. (2019) reported a negative relationship between δ^13^C and δ^15^N and a positive correlation between δ^13^C and N concentration of leaf tissue. In the present study, δ^13^C and δ^15^N of shoot biomass were positively correlated in 1 year but not the other. A positive correlation between these two phenotypes indicates that genotypes with higher WUE derived smaller amounts of N from SNF than those with lower WUE. However, the fact that these two traits were not correlated in the first year indicates that breeding for high WUE, as well as high N fixation, is not mutually exclusive. In fact, given the high sensitivity of soybean SNF to drought (Sinclair et al., 1987; Purcell et al., 2004; King et al., 2014), high WUE could be advantageous for SNF in some environments, which is suggested by the negative correlation between δ^13^C and δ^15^N that were observed by Steketee et al. (2019). This is also consistent with observations by Kumarasinghe et al. (1992), who reported a positive correlation between WUE efficiency and SNF based on C and N isotope measurements. Similar to the relationships between δ^13^C and δ^15^N, correlations between δ^13^C and [N] and between δ^13^C and C/N were significant only in 1 year but not the other. Given the significant environment and genotype x environment interactions for these traits, differences between the 2 years with respect to the relationships between these traits were not surprising.

### 4.2 Population structure and marker-trait associations and candidate genes

Whole-genome resequencing data for all the genotypes used in this study provided much more abundant SNP markers (~4.1 million) to identify genomic regions associated with δ^13^C, δ^15^N, [N], and C/N ratio than previous studies that used genotypic data from the SoySNP50K iSelect SNP Beadchip (Dhanapal et al., 2015b; Kaler et al., 2017; Steketee et al., 2019). However, the goal of using SNPs derived from whole-genome resequencing data, coupled with the restriction based on maturity, resulted in a more limited number of entries for this study (105 entries) compared to previous GWAS studies (211-373 entries) examining these traits in soybean (Dhanapal et al., 2015a; Dhanapal et al., 2015b; Kaler et al., 2017; Steketee et al., 2019). Despite the lower number of entries than earlier GWA studies of these traits, the genetic diversity within the current panel was high and PC analysis used to assess population structure revealed eight subpopulations within the panel (**Figure 3**), which is similar to the six to nine subpopulations used by others for soybean GWA studies using whole-genome resequencing data or SoySNP50K iSelect SNP Beadchip data (Li et al., 2008; Dhanapal et al., 2015a; Dhanapal et al., 2015b; Contreras-Soto et al., 2017; Wang et al., 2018; Boudhrioua et al., 2020; Seck et al., 2020). In the current study, cutoff *p*-value thresholds to declare a marker-trait association significant were more stringent compared to other soybean GWA studies (Dhanapal et al., 2015a; Dhanapal et al., 2015b; Kaler et al., 2017; Zhang et al., 2018; Fang et al., 2020; Kaler et al., 2020; Song et al., 2020). Based on the applied thresholds, we identified a total of 104 significant marker-trait associations, which tagged 76 putative loci for the four traits. Although most of these loci were novel, several of them overlap or are located within close physical distances of loci previously reported for the four traits.

#### 4.2.1 Carbon isotope ratio

GWA mapping of δ^13^C identified a total of 11 loci, 10 of which were marked by single SNPs and one that was marked by three SNPs (**Table 3** and **Supplementary Table S6**). Among these loci, six were novel, whereas five were closely located to previously identified markers for δ^13^C by (Dhanapal et al., 2015a; Kaler et al., 2017; Steketee et al., 2019). These five loci (**Table 3** and **Supplementary Table S6**) underscore the importance of these genomic regions relative to soybean WUE in different environmental conditions, and the application of orders of magnitude higher marker density than those used in previous studies may identify markers closer to candidate genes. Among the novel loci, locus 7 on Gm13 was identified in both years and had the greatest marker effect (−0.34, **Table 3** and **Supplementary Table S6**) among the identified δ^13^C loci. Exploration of the gene models in the vicinity of novel and confirmed loci revealed several genes with functions associated with stomatal morphogenesis and function (**Table 3** and **Supplementary Tables S7** and **S8**). Although not much is known about some of the gene models, for others, evidence that they affect plant responses to water availability, transpirational water loss, and/or WUE is available in the literature. A total of five candidate genes were identified at locus 7 based on keyword searches. Glyma.13G028200, encoding *Photosystem II Reaction Center Protein A* (*PsbA*), when overexpressed in maize (*Zea mays*), resulted in a much lower reduction in net photosynthesis and stomatal conductance than in wild-type plants under drought conditions (Huo et al., 2016). Another candidate gene, Glyma.13G030900, encoding *NAC* domain transcription factor *ATAF1*, is expressed in different tissues in Arabidopsis, including in stomatal guard cells (Lu et al., 2007). Wu et al. (2009) showed that *ATAF1* overexpressing plants treated with ABA exhibited a greater extent of stomatal closure compared to *ataf1* mutant and wild types, indicating *ATAF1* plays a role in ABA-mediated stomatal closure to reduce water loss. *SPEECHLESS (SPCH)* (Glyma.13G040100), another locus-7 candidate gene, can control the stomatal number and density by initiating asymmetric cell divisions to establish stomatal lineage in Arabidopsis(MacAlister et al., 2006). Tripathi et al. (2016) showed that *SPCH* and its two other co-orthologues had reduced mRNA expression in drought-induced soybean leaves, resulting in reduced stomatal density (SD) and stomatal index compared to the untressed soybean leaves.

Locus 2 on Gm02, anchored by three SNPs, is particularly interesting as both Dhanapal et al., 2015a and Kaler et al. (2017) reported QTL for δ^13^C close to this locus. Additionally, Kumar and Lal (2015) also found one highly polymorphic SSR marker close to this locus after testing several SSR markers for polymorphism between soybean genotypes with varying CID. However, only one gene model (Glyma.02G113800) was identified as a hypothetical protein, and a GO annotation of “stomatal complex morphogenesis” was identified within this locus based on keyword searches and allele combinations.

Steketee et al. (2019) reported significant loci for δ^13^C within 1.5 Mb upstream or downstream of loci 6, 8, and 10 of the current study. Although no candidate genes were identified in the vicinity of locus 6, five were identified at locus 9, and one at locus 10. Two of the genes at locus 9 encode a *CBL-interacting protein kinase 2* (*CIPK2*; Glyma.14G202700) and a *CBL-interacting protein kinase 11* (*CIPK11*; Glyma.14G203000). These genes are annotated with GO biological processes like stomatal movement and ABA signaling. Overexpression of *CIPK2* in tobacco, Arabidopsis, and soybean has been reported to confer drought tolerance in control environment studies by modulating stomatal closure through ABA signaling under drought stress (Wang et al., 2016; Xu et al., 2021). On the other hand, overexpression of *CIPK11* confers drought sensitivity in transgenic Arabidopsis (Ma et al., 2019). Further experiments by Ma et al. (2019) suggest that *CIPK11*-mediated plant response to drought tolerance is dependent on Di19-(dehydration-induced19-3), a transcriptional activator known to be involved in modulating plant response to different abiotic stresses, including drought stress. Another candidate gene at locus 9, Glyma.14G202900, encodes *CHAL* or *EPFL-6*, which is a homologue of *Epidermal Patterning Factor-1* (*EPF1*) (Abrash and Bergmann, 2010). Overexpression of *EPF1* in rice significantly reduces the SD and anatomical maximum stomatal conductance, leading to reduced water consumption and higher intrinsic WUE (Caine et al., 2019; Mohammed et al., 2019; Caine et al., 2023). The identified candidate gene models are targets for further studies that are needed to ascertain the extent of involvement, or lack thereof, of these candidate genes (**Table 3** and **Supplementary Tables S7** and **S8**) in soybean WUE.

#### 4.2.2 Nitrogen isotope ratio

The extent of SNF can be assessed under field conditions using N stable isotopes. Nitrogen isotope ratio (δ^15^N) of plant tissue is commonly used to determine relative SNF expressed as %Ndfa. Nitrogen derived from the atmosphere is computed by relating the δ^15^N value of a genotype to the δ^15^N value of a non-nodulating reference line (Kohl et al., 1980). However, δ^15^N can also be used directly to assess relative SNF if non-nodulating reference lines are unavailable because the δ^15^N of the reference non-nodulated line is often constant across the field (Peoples et al., 2002; Steketee et al., 2019; Bazzer et al., 2020c). Here, association analysis based on δ^15^N identified 22 loci on 16 different chromosomes. Four of these loci were detected in both environments, indicating the stability of these loci despite significant environmental effects between the environments (**Table 3** and **Supplementary Table S6**). Additionally, three of the loci, including one of those identified in both environments (locus 13), marked genomic regions previously identified by Steketee et al. (2019). Interestingly, no candidate genes were identified in the genomic regions marked by these loci, neither based on keyword searches nor based on allelic combination analyses. However, four candidate genes were identified in the vicinity of novel δ^15^N loci 4, 7, 8, and 21. Among the four gene models, Glyma.08G152700 at locus 8 is annotated as an *AGAMOUS-like 26* (*AGL-26*) homologue, which, in Arabidopsis, is responsive to N deprivation and re-supply (Gan et al., 2005). *AGAMOUS-like* transcription factors also appear to be involved in regulating the symbiotic relationship between the plant and rhizobia in common bean (*Phaseolus vulgaris*) (Ayra et al., 2021).

Similarly intriguing is the potential role of Importin α nuclear transport receptors (Harreman and Adam, 2004), which may be involved in nodulation processes based on GO biological functions annotation. This suggests that the *Importin* α *isoform 4* homologue identified at locus 21 may play a role in nodulation in soybean. Additional studies aimed at identifying the regulatory regions associated with the identified loci are critical to better understand and improve SNF in soybean. Of particular interest for follow-up, studies are the loci that were consistent across the environments as well as those that overlapped with previously identified regions of the soybean genome (**Table 3** and **Supplementary Table S6**). Among these, loci 3 and 22 are highly significant as they both possess very high logarithm of odd ratio (**Table 3** and **Supplementary Table S6**) in both environments compared to our cutoff values (**Supplementary Table S4**). These two loci also exhibit the largest marker effects compared to all other significant markers (**Table 3** and **Supplementary Table S6**), but, interestingly, no candidate genes were identified in the vicinity of either of these loci.

#### 4.2.3 N concentration

Twenty-one loci were detected for shoot [N] in this study; among them, loci 3 and 19 are located in the proximity of previously detected loci for leaf [N] by Steketee et al. (2019), one locus (17) coincided with a shoot [N] locus previously detected by Dhanapal et al. (2015b). Furthermore, loci 3, 9, 11, and 17 were near significant markers identified for shoot ureide concentration by Ray et al. (2015) (**Table 3** and **Supplementary Table S6**). This is of interest because ureides (allantoin and allantoate) are the N-rich products from N_2_-fixation that are transported throughout the plant and may be used as indicators of the sensitivity of N_2_-fixation to drought stresses in common bean and soybean (Sinclair et al., 2000; Vadez and Sinclair, 2001; King and Purcell, 2005; Coleto et al., 2014). Even though based on analysis of [N] in different plant tissue (leaf vs. whole shoot; Steketee et al., 2019) or a different N-related trait (ureide concentration), the coincidence of loci between these studies is intriguing and warrants further attention. Therefore, the five loci associated with both shoot [N] and ureide concentration may be valuable to breed for sustained N_2_ fixation under water-limited conditions.

Several candidate genes were identified in the vicinity of locus 19, which was tagged for [N] in both environments and by Steketee et al. (2019). This includes a gene model (Glyma.16G156700) identified by keyword search that encodes a *SCARECROW* (*SCR*) homologue. In several legumes, including soybean, *SCR* is expressed in root cortical cells, and, in *Medicago truncatula*, nodule number and density are reduced in *SCR* mutants (Dong et al., 2020). Based on allelic comparisons at locus 19, several transporters, as well as two transcription factors were included in the candidate gene list (**Table 3** and **Supplementary Table S8**). This included a GATA transcription factor (Glyma.16g155300), numerous of which have been shown to be responsive to N availability, and two that were implicated to be involved in the regulation of soybean N metabolism (Zhang et al., 2015). An *Ammonium transporter 2* (*AMT2*) homologue (Glyma.02G043700) was identified close to the SNP that anchored the novel [N] locus 2. In Arabidopsis, *AMT2.1* contributes to ammonium uptake in roots and functions mainly in root-to-shoot translocation of ammonium under N deficiency conditions (Giehl et al., 2017). As the N status of soybean is a function of both SNF and uptake of mineral N from the soil, it is not surprising that candidate genes associated with both processes were identified within the LD regions of loci associated with shoot [N]. Indeed, it appears that breeding for greater yield has enhanced the ability of soybean to acquire more N from both sources (Donahue et al., 2020).

#### 4.2.4 Carbon nitrogen ratio

The relationships among C assimilation, N uptake, and SNF are closely regulated. Photosynthetic output can be negatively affected due to N deficiency but can recover with N supplements (Coruzzi and Bush, 2001; Coruzzi and Zhou, 2001). On the other hand, increased C supply can improve N uptake and metabolism (Coruzzi and Bush, 2001; Coruzzi and Zhou, 2001). In a hydroponic study, Bacanamwo and Harper (1996) found that the shoot C/N ratio was positively correlated with the activity of nitrogenase in soybean nodules and suggested that regulation of nitrogenase activity entails tissue C as well as N concentrations. The balance between C and N assimilation in soybean also is influenced by the negative impact of drought, which is more severe on SNF than on leaf photosynthesis, and partial recovery from prolonged drought is slower for SNF than for photosynthesis (Djekoun and Planchon, 1991). Significant genotypic variation in the C/N ratio exists in soybean, but, to our knowledge, only one study has identified molecular markers for this trait. Dhanapal et al. (2015b) used GWAS to identify 17 putative C/N ratio loci in MGIV soybean grown in multiple field environments. Interestingly, none of the 22 loci associated with C/N in the present study overlapped with any of those reported by Dhanapal et al. (2015b) (**Table 3** and **Supplementary Table S6**). The absence of overlap with loci identified by Dhanapal et al. (2015b) was unexpected, particularly in light of the coincidence in loci for shoot [N] with previous studies and the detection of three loci (5, 9, and 19) in both environments of the present study, but likely is due to factors such as complexity of the regulation of C and N uptake and assimilation, particularly under different environmental conditions, as well as the makeup of the diversity panels used in the two studies. Not surprisingly, given the strong correlation between the two traits (**Table 2**), five C/N-associated loci (1, 6, 11, 19, and 21) colocalized with [N]-associated loci in the current study. Naturally, this also led to an overlap in gene models of interest for locus 19 (the only locus among these five for which candidate genes were identified). Among the candidate genes identified at other C/N loci, Glyma.08G118500 encoding *C-terminally Encoded Peptide 14 (CEP14)* was identified based both on keyword search and on the allelic combination approach. The anchoring marker of locus 10 is located within the coding region of *CEP14*, which also is within about 600 Kb of the δ^15^N locus 7 and [N] locus 12. In Arabidopsis, under N-limitation, *CEP1* serves as a root-to-shoot signal that is perceived by *CEP* receptors in the shoot which leads to shoot-to-root signaling that causes an upregulation of nitrate transporter genes in roots (Tabata et al., 2014). *CEP*s are also involved in the regulation of nodule numbers in legumes, where *CEP*s positively regulate nodulation (Imin et al., 2013; Laffont et al., 2020). Several *CEP*s, including *CEP14*, are regulated by environmental cues such as N depletion and increased CO_2_ levels in shoots and roots of Arabidopsis (Delay et al., 2013), as was observed for *CEP1* in Medicago (Imin et al., 2013). Further studies are required to establish whether *CEP14* is causal for the association of locus 10 with C/N, but its involvement in the signaling of N conditions and responsiveness to CO_2_ documented in the literature underscores its potential as a target to develop a better understanding of the regulation of C and N status in soybean.

## 5 Conclusion

Genomwide association analysis was conducted for δ^13^C, δ^15^N, [N], and C/N with 105 genotypes and a high-density marker panel consisting of more than 4.1 million SNPs derived through whole-genome resequencing. A total of 76 genomic loci associated with these traits were detected, namely, 11 for δ^13^C, 22 for δ^15^N, 21 for [N], and 22 for C/N. Nine loci associated with different traits were consistent across the two environments. In addition, several loci were associated with more than one trait, including four loci for [N] and C/N, and one locus for δ^15^N and C/N and, consequently, may serve as markers for multiple traits. Although most identified loci were novel, 14 aligned with previously detected genomic regions for the same or similar traits. Exploration of the gene models in the vicinity of the loci identified 59 candidate genes. Further scrutiny of these candidate genes will facilitate a better understanding of WUE, SNF, and N and C dynamics. The genomic regions and candidate genes identified in this study may serve to breed for soybean with enhanced WUE and improved and sustained SNF and to further dissect the mechanisms controlling WUE and SNF.

## Data availability statement

The raw data supporting the conclusions of this article will be made available by the authors, without undue reservation.

## Author contributions

MA: Data curation, Formal analysis, Writing – original draft, Writing – review & editing. SM: Data curation, Formal analysis, Writing – review & editing. AS-S: Investigation, Writing – review & editing. HZ: Investigation, Writing – review & editing. AS: Conceptualization, Writing – review & editing. FF: Conceptualization, Funding acquisition, Writing – original draft, Writing – review & editing.
